# Ste20-Related Proline/Alanine-Rich Kinase (SPAK) Regulated Transcriptionally by Hyperosmolarity Is Involved in Intestinal Barrier Function

**DOI:** 10.1371/journal.pone.0005049

**Published:** 2009-04-03

**Authors:** Yutao Yan, Guillaume Dalmasso, Hang Thi Thu Nguyen, Tracy S. Obertone, Shanthi V. Sitaraman, Didier Merlin

**Affiliations:** Department of Medicine, Division of Digestive Diseases, Emory University School of Medicine, Atlanta, Georgia, United States of America; Charité-Universitätsmedizin Berlin, Germany

## Abstract

The Ste20-related protein proline/alanine-rich kinase (SPAK) plays important roles in cellular functions such as cell differentiation and regulation of chloride transport, but its roles in pathogenesis of intestinal inflammation remain largely unknown. Here we report significantly increased SPAK expression levels in hyperosmotic environments, such as mucosal biopsy samples from patients with Crohn's disease, as well as colon tissues of C57BL/6 mice and Caco2-BBE cells treated with hyperosmotic medium. NF-κB and Sp1-binding sites in the SPAK TATA-less promoter are essential for SPAK mRNA transcription. Hyperosmolarity increases the ability of NF-κB and Sp1 to bind to their binding sites. Knock-down of either NF-κB or Sp1 by siRNA reduces the hyperosmolarity-induced SPAK expression levels. Furthermore, expression of NF-κB, but not Sp1, was upregulated by hyperosmolarity *in vivo* and *in vitro*. Nuclear run-on assays showed that hyperosmolarity increases SPAK expression levels at the transcriptional level, without affecting SPAK mRNA stability. Knockdown of SPAK expression by siRNA or overexpression of SPAK in cells and transgenic mice shows that SPAK is involved in intestinal permeability *in vitro* and *in vivo*. Together, our data suggest that SPAK, the transcription of which is regulated by hyperosmolarity, plays an important role in epithelial barrier function.

## Introduction

Inflammatory bowel diseases (IBD), including ulcerative colitis (UC) and Crohn's disease (CD), are multi-factorial diseases typically associated with relapsing diarrhea, which is caused by increased paracellular permeability of the intestinal epithelial lining and an intestinal hyperosmotic environment [1], [2], [3], [4], [5]. Intestinal epithelial cells (IECs) are exposed to the second most extreme osmotic environment after kidney. In many forms of IBD, including CD, neonatal necrotizing enterocolitis and UC, this extreme hyperosmolarity contributes to the exacerbation of intestinal inflammation via upregulation of inflammatory molecules such as matrix metalloproteinase (MMP)-9 [6], epithelial cytokine response-interleukin (IL)-8 [7], [8], [9], [10], IL-1 [11], [12], [13], and tumor necrosis factor (TNF)-α [13], downregulation of vascular cell adhesion molecule (VCAM)-1 [14], or methylation of protein phosphatase 2A [15]. Thus, hyperosmolarity is recognized as a proinflammatory signal [12], [16] in addition to classic inflammatory signals including bacteria, bacterial byproducts or proinflammatory cytokines. In addition to IECs, osmotic stress presents an important challenge to normal cell function in a variety of other cells, including peripheral blood mononuclear cells [7], human bronchial epithelial cells [8], [9] and the corneal epithelium [17]. Hyperosmolarity has been proposed to play a role in intestinal inflammation in several inflammatory bowel diseases, including CD and UC, as well as newborn and neonatal necrotizing enterocolitis [1], [2], [3], [4], [5].

Yeast ste20 kinases, and the mammalian homologs p21-activated kinase (PAK) and germinal center kinase (GCK), function as mitogen-activated protein kinase kinase kinase kinases (MAP4K) and have central and well-described roles in “cell-volume sensing” and in regulating a wide variety of gene functions, including barrier-related functions [18], [19], [20], [21], [22]. Lymphocyte-oriented kinase (LOK, ste20-like kinase) [23] and Ste20-like kinase (SLK) [24] can regulate actin cytoskeleton reorganization during cell adhesion and spreading, whereas PAK increases endothelial permeability [25], [26].

The ste20-like proline/alanine-rich kinase (SPAK) belongs to the GCK IV subfamily, members of which contain an N-terminal series of proline and alanine repeats (PAPA box), followed by a catalytic domain, a nuclear localization signal, a consensus caspase-cleavage motif, and a C-terminal regulatory region [27], with the missing PAPA box and F-alpha helix loop present in its colonic isoform [28], [29]. SPAK plays roles in cell differentiation [27], [30], cell transformation and proliferation [31], regulation of chloride transport [32], [33], [34], and mediation of intestinal inflammation [29]. Specifically, SPAK can phosphorylate Na^+^-K^+^-2Cl^−^ cotransporter 1 (NKCC1), which has an important role in inflammation [35], [36] and maintaining intracellular and extracellular ion homeostasis [37]. Recently, we have demonstrated that under inflammatory conditions, TNF-α is a key regulator of SPAK expression [29].

The relationships among hyperosmolarity, SPAK and IBD are unknown. A better knowledge of these relationships will further our understanding of IBD physiopathology. The present study was therefore undertaken to determine how SPAK expression is regulated by hyperosmolarity and investigate its involvement in epithelial barrier function loss, which occurs during IBD.

## Results

### Colonic SPAK is upregulated in patients with CD

**Figure 1:**
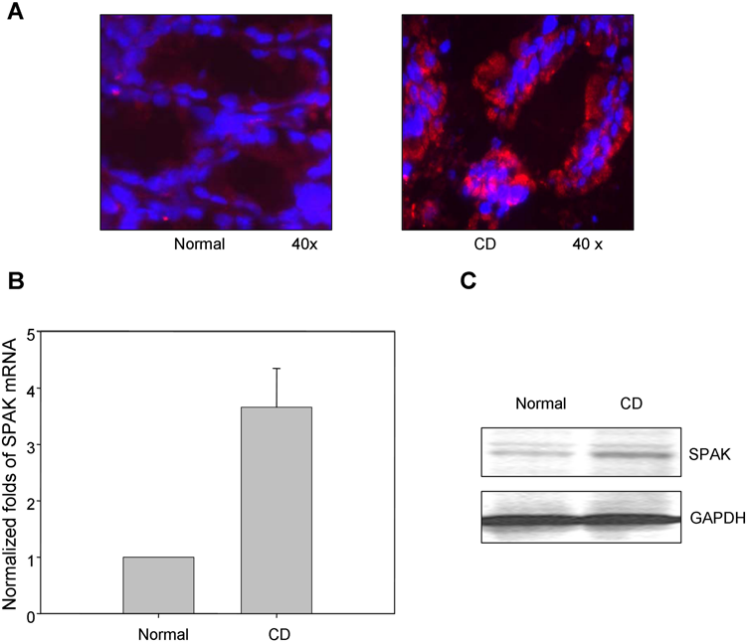
SPAK expression profile in colon tissue from patients with ulcerative colitis. A. Immunostaining of SPAK in normal human colon tissue and Crohn's disease (CD) patient colon tissue from mucosal biopsies. SPAK expression (red); nuclear staining by DAPI (blue); SPAK is primarily expressed in epithelial cells. B. The expression of SPAK mRNA in normal human and Crohn's disease (CD) patient colon tissues from mucosal biopsies were quantified by real-time PCR, ** p<0.01. C. 30 μg of protein from normal human colon and Crohn's disease (CD) patient colon from mucosal biopsies were examined by western blot with SPAK antibody, colon tissue from CD patients demonstrated significantly higher levels of SPAK expression (upper part) vs. healthy colon, with GAPDH as the internal loading control.

As a preliminary step in a functional study of SPAK in IBD, it is sensible to study its expression profile. We examined SPAK expression in colonic biopsy samples from healthy and CD patients. Immunofluorescence of biopsy specimens from CD patients showed increased colonic SPAK expression, mainly in epithelial cells, compared with specimens of healthy colon tissue (Fig. 1A). Real-time PCR and Western-blot analyses showed that colonic mucosa from the same CD patients contained significantly increased SPAK expression at both the mRNA and protein levels compared with tissue from normal subjects (Fig. 1B and 1C).

### Expression of SPAK is enhanced in colon tissue from hyperosmolarity treated mice

**Figure 2:**
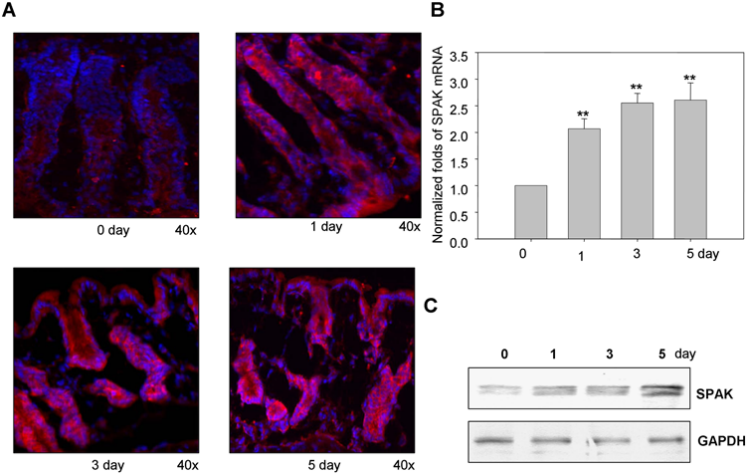
SPAK expression profile in colon tissue from mice treated with hyperosmolarity. A. Immunostaining of SPAK in colon sections of mice treated with hyperosmolarity at 0, 1, 3, and 5 days. SPAK (red); nuclear staining by DAPI (blue). B. Real time PCR analysis of SPAK mRNA expression in mucosa from colon tissue of hyperosmolarity treated mice. ** p<0.01. C. Western blot analysis of SPAK (upper part) expression in mucosa from colon tissue of hyperosmolarity treated mice, with GAPDH as the internal loading control.

Next we examined the relative SPAK expression levels in colon samples from healthy mice and mice exposed to hyperosmotic conditions. Immunofluorescence analysis showed increased levels of colonic SPAK expression, mainly in epithelial cells, compared with sections from untreated colonic tissue (Fig. 2A). As shown in Fig. 1A and 2A, hyperosmolarity induces cell-wall damage and tissue shrinkage, and increases the space between crypts. Epithelial damage is apparent as early as 1 day after exposure to hyperosmolarity and progressively increases to a maximum level at day 5. Correspondingly, SPAK mRNA (Fig. 2B) and protein (Fig. 2C) levels in colonic tissue increase under hyperosmotic conditions and correlate with the extent and duration of colonic injury (Fig. 2A). The greatest increases in SPAK expression levels were on days 3 and 5; SPAK transcripts were detected at approximately 2.7-fold greater levels compared with untreated mice (Fig. 2B), as shown by real-time PCR, immunohistochemistry and Western-blot analyses. Together, these results indicate a strong correlation between SPAK expression level and the extent of hyperosmolarity.

### Hyperosmolarity increases SPAK expression in intestinal epithelial cell lines *in vitro*

**Figure 3:**
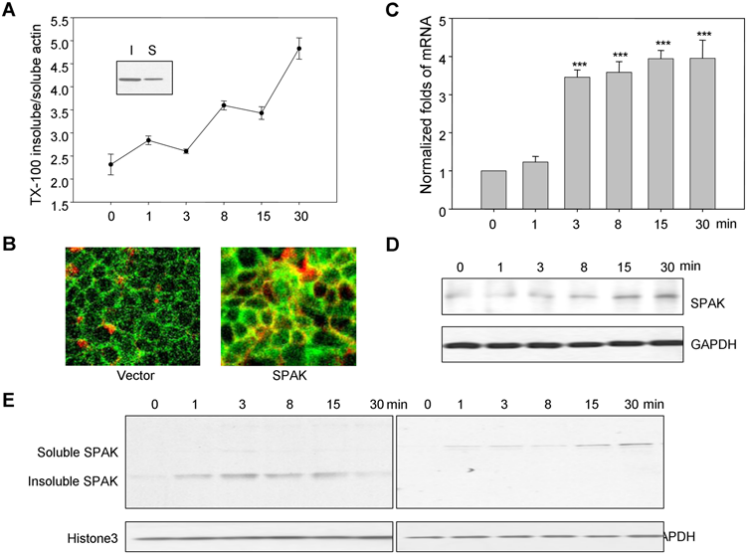
SPAK expression in hyperosmolarity treated Caco2-BBE cells. A. Graph of ratio of triton x-100 insoluble actin *v.s.* soluble actin and western blot. B. Immunostaining of SPAK in colonic Caco2-BBE cells treated with hyperosmolarity at 0 and 15 min. SPAK (green); actin by rhodamine (red). (C) Real time PCR and (D) Western blot demonstrated that treatment of hyperosmolarity increases SPAK expression with GAPDH as internal loading control, ** p<0.01, *** p<0.001. E. Western blot showed that SPAK expression is increased by hyperosmolarity and is recruited to the triton-100 insoluble pool at 0, 1, 3, 8, 15 and 30 min.

Actin plays an important role in cell shape, volume and regulation of barrier function through interaction with tight-junction proteins; hence we examined the effects of hyperosmolarity on distribution of actin and expression levels of SPAK. We found that hyperosmolarity treatment leads to increased levels of Triton x-100-insolube F-actin and an increased ratio of F-actin versus G-actin (Fig. 3A). The newly polymerized F-actin predominantly localizes to the plasma membrane, where it forms a thick ring [38], [39] that persists as long as hyperosmolarity is maintained. Furthermore, immunofluorescence showed that hyperosmotic treatment leads to Caco2-BBE cell shrinkage and increases the intercellular space. Importantly, hyperosmotic conditions significantly increased SPAK expression levels and recruitment of SPAK to the membranes of cells (Fig. 3A). SPAK mRNA and protein levels were also increased, reaching maximum level as early as 3 min after hyperosmotic treatment (Fig. 3B, 3C and 3D). Here, we cannot conclude whether the high SPAK level was due to new synthesis or recruitment of SPAK to the membrane, or both. Under hyperosmotic conditions, SPAK levels increase in Triton x-100-soluble and insoluble pool, indicating SPAK recruitment to the F-actin-associated pool (Fig. 3E). Together, these data show that, under hyperosmotic conditions, increased SPAK is redistributed to actin-containing regions of the plasma membrane.

### Induction of SPAK is primarily at the transcriptional level

**Figure 4:**
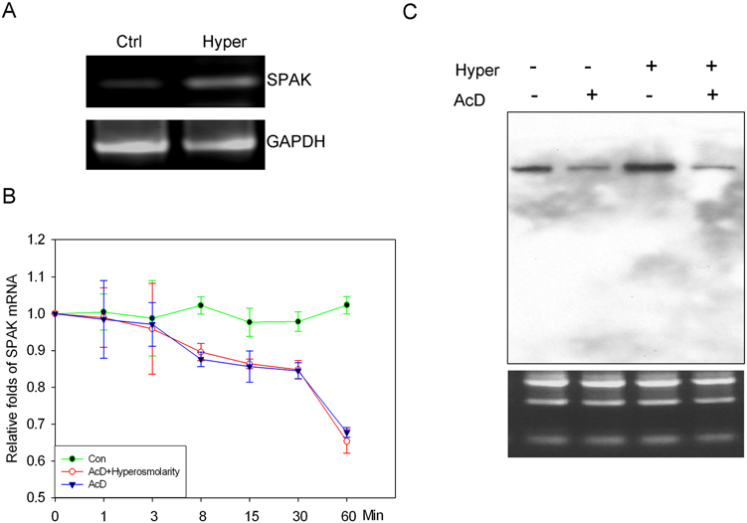
Hyperosmolarity regulates SPAK expression at the transcriptional level. A. Nuclear run-on assay indicated the increase of SPAK mRNA transcription under the treatment of hyperosmolarity, with the mRNA transcription of GAPDH as internal control. B. Hyperosmolarity does not change SPAK mRNA stability; the percentage of remaining SPAK mRNA is shown at the different time point. Solid circle represents the value of real time PCR with the samples from Caco2-BBE cells without treatment, open circle represents the value of real time PCR with the samples from Caco2-BBE treated with actinomycin D, solid triangle represents real time PCR with the samples from Caco2-BBE treated with actinomycin D and hyperosmolarity. C. Northern blot analysis of total RNA from Caco2-BBE cells, Lane 1, no treatment, Lane 2, AcD, Lane 3, hyperosmolarity, and Lane 4, AcD and hyperosmolarity. The lower RNA electrophesis shows equal loading of each condition.

To determine whether the induction of SPAK expression under hyperosmotic conditions was mediated by transcriptional or post-transcriptional mechanisms, nuclear run-on assays (Fig. 4A) were performed in Caco2-BBE cells pretreated with or without hyperosmotic conditions. Compared with nuclei not exposed to hyperosmotic conditions, exposure to hyperosmotic conditions increased SPAK mRNA levels. This experiment was repeated three times and similar results were obtained each time. These results indicate that this increase in SPAK levels is due to increased transcription.

### The increase of SPAK transcription by hyperosmolarity is not mediated by alteration of mRNA stability

Changes in steady-state mRNA levels may be due to changes in the degradation rate of a transcript and/or rate of gene transcription. Hence, it was important to investigate the relative contribution of post-transcriptional mechanisms in the modulation of SPAK mRNA levels by hyperosmolarity. To assess SPAK mRNA stability, Caco2-BBE cells were treated with 5 μg/ml of AcD to inhibit mRNA synthesis, and SPAK mRNA levels were measured at the indicated time points in the presence and absence of hyperosmolarity by real-time RT-PCR; 18S rRNA was used as an internal control to normalize SPAK mRNA levels. The decay rate of SPAK mRNA in AcD-treated Caco2-BBE cells was almost the same as that of SPAK mRNA in cells treated with AcD plus hyperosmolarity; no significant difference was detected (Fig. 4B). In parallel, Northern-blot analyses were performed on 20-μg samples of Caco2-BBE mRNA treated with or without AcD (5 μg/ml) and/or exposed to hyperosmotic conditions (Fig. 4C). Results showed that AcD can significantly reduce mRNA levels (Fig. 4C lane 2) compared with untreated Caco2-BBE cells (Fig. 4C lane 1). In addition, hyperosmolarity treatment (in the absence of AcD treatment) markedly increased levels of SPAK mRNA transcripts. However, if Caco2-BBE cells were pretreated with AcD for 1 hour, hyperosmolarity cannot reverse the AcD-induced mRNA degradation. Together, these findings indicate that the observed changes in SPAK protein level are due to increased SPAK mRNA transcription rather than changes in mRNA stability.

### Sp1- and NF-κB-binding sites have roles in SPAK promoter activity

**Figure 5:**
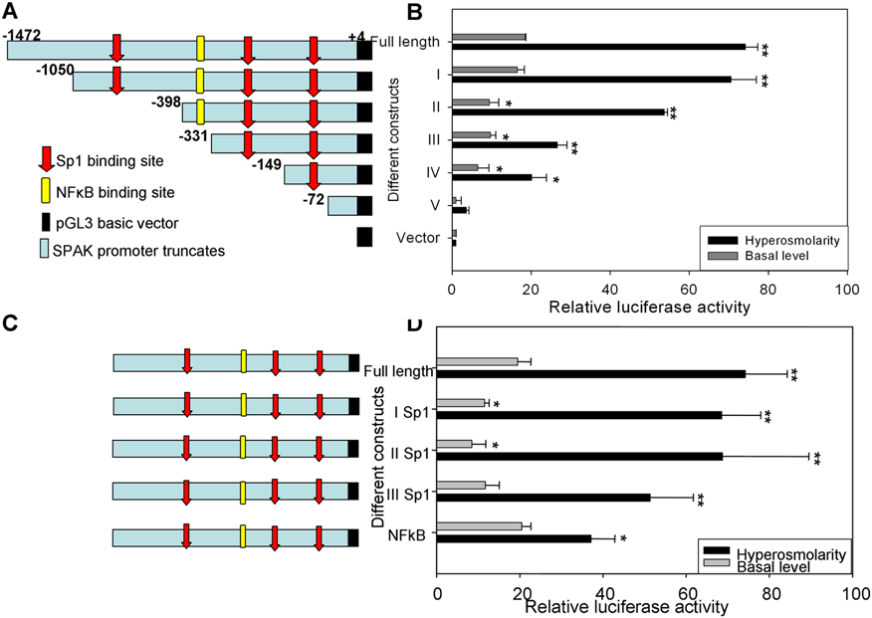
Characterization of SPAK promoter. A. Schematic representation of human SPAK promoter constructs. the full-length SPAK promoter (nt-1472 to +4); construct I (nt −1050 to +4); construct II (nt −398 to +4); construct III (nt −331 to +4); construct IV (nt −149 to +4) and construct V (nt −72 to +4). Numbers are given in relation to the translational start codon (+1) and indicate 5′-ends of the deletion constructs. The location of the identified positive regulatory region is indicated by a light blue box. Positions of the putative Sp1 (Red) and NF-κB (Yellow) sites are indicated by arrows. B. Promoter activities of the 5′ deleted constructs in un-treated or hyperosmolarity-stimulated Caco2-BBE cells normalized to *Renilla* Luc activities driven by the phRL-CMV control vector. Activities are expressed as fold inductions over cells transfected with the empty pGL3-basic vector. Each value represents the mean±SD of at least 3 independent sets of transfection experiments performed in triplicate, *p<0.05; **p<0.01. C. Schematic representation of mutated SPAK promoter constructs: the full-length SPAK promoter; I Sp1 binding site (−496); II Sp1 binding site (−303); III Sp1 binding site (−114) and NF-κB binding site (−354). The digits are given in relation to the translational start codon (+1). The location of the identified positive regulatory region is indicated by a light blue box. Positions of the putative Sp1 sites are indicated by arrows and NF-κB is indicated by rectangle. The corresponding mutated transcription factor binding site is indicated by black arrow or black rectangle. D. Effects of mutations of Sp1 or NF-κB binding sites on SPAK promoter activity. The various mutated constructs were transiently transfected into Caco2-BBE cells under the basal (gray bar) or hyperosmolarity conditions. Promoter activity of the full-length wild-type construct was set to 100% (control). Values represent means±SD of at least 3 independent sets of transfection experiments performed in triplicate, *p<0.05, **p<0.01.

Having demonstrated that hyperosmolarity-induced SPAK expression is regulated at the transcriptional level, we cloned a ∼1.5-kb fragment of the 5′-flanking region of the SPAK gene from human genomic DNA to further understand the molecular mechanisms underlying the increased expression of SPAK. To identify the core promoter region of the SPAK gene, we generated five partial deletion constructs fused with the luciferase reporter gene. Caco2-BBE cells were transiently transfected with these constructs, then stimulated by exposure to hyperosmotic conditions (610 mOsm) for 30 min. Transcriptional activities were then measured using the Dual-Luciferase Reporter Assay System (Promega, San Luis, CA). The full-length promoter displayed increases of ∼18-fold in basal promoter activity and 74-fold in hyperosmolarity-stimulated promoter activities, compared with the empty pGL3 basic vector (Fig. 5A and 5B). Constructs I, II, III, IV and V had ∼16, 9, 10, 6 and 1-fold greater promoter activities, respectively, compared with the pGL3 vector at the basal level. Under hyperosmotic conditions, constructs I and II showed ∼70 and 53-fold increases, respectively, in promoter activity compared with the basic vector pGL3, to give activities that were about 95% and 72%, respectively, of the activity of the full-length SPAK promoter. The basal and hyperosmolarity-induced promoter activities of constructs III, IV and V were greater than those of the pGL3 vector (∼26, 20, 4-fold increases, respectively); however, the activities were only about 35%, 27% and 6%, respectively, of that of the full-length SPAK promoter (Fig. 5B). To investigate and confirm the functional roles of the relevant binding motifs in regulating SPAK promoter activity, we generated various Sp1- and NF-κB-binding mutants (Fig. 5C). Using the transcriptional activity assay, we found that all the Sp1 mutants had significantly reduced basal promoter activities compared with the wild-type promoter, and the hyperosmolarity-stimulated activity levels of these Sp1 mutants were also lower than the wild-type promoter activity (Fig. 5C and 5D). By contrast, although the NF-κB mutant basal luciferase activity was similar to that of the wild-type promoter, there was a marked reduction in hyperosmolarity-stimulated promoter activity (∼50% reduction) (Fig. 5D). Taken together, these results show that the Sp1-binding sites are important in basal and stimulated SPAK promoter activities, and that the NF-κB-binding site has a crucial role in hyperosmolarity-stimulation of SPAK promoter activities.

### Sp1 and NF-κB are physically associated with their corresponding binding sites

**Figure 6:**
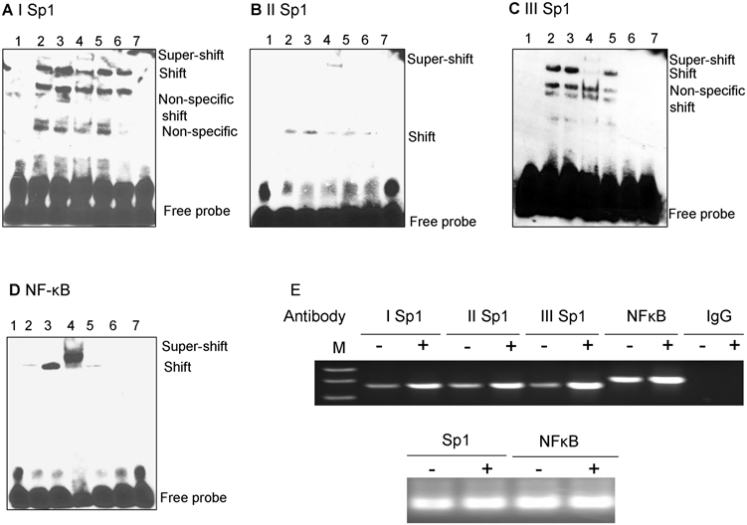
EMSA of (A) I Sp1 (−496), (B) II Sp1 (−303), (C) III Sp1 (−114), (D) NF-κB (−354). Lane 1, biotin-labeled oligonucleotide alone; lane 2, biotin-labeled oligonucleotides incubated with 5 μg Caco2-BBE nuclear extracts; lane 3, biotin-labeled oligonucleotides incubated with 5 μg hyperosmolarity-treated Caco2-BBE nuclear extracts; lane 4, biotin-labeled oligonucleotides incubated with 5 μg Caco2-BBE nuclear extracts in the presence of anti-Sp1 (A–C) or NF-kB (p65) (D) antibodies; lane 5, biotin-labeled oligonucleotides incubated with 5 μg Caco2-BBE nuclear extracts in the presence of non-specific IgG; lane 6, biotin-labeled oligonucleotides incubated with 5 μg Caco2-BBE nuclear extracts in the presence of a 50-fold excess of cold competitor oligonucleotide; lane 7, biotin-labeled binding site-mutated oligonucleotides incubated with 5 μg Caco2-BBE nuclear extracts. E. Chromatin immunoprecipitation (ChIP) assay: the antibodies indicated were incubated with cross-linked DNA isolated from Caco2-BBE cells treated with (+) or without (−) hyperosmolarity, IgG antisera acts as control. Sp1 (I, II, and III) and NF-κB promoter elements in the immunoprecipitates were detected by PCR. The lower panel shows DNA input as template for internal control.

To study the association between the transcription factors and the corresponding binding sequences, and to further confirm the importance of Sp1 and NF-κB in activation of the SPAK gene, we used electrophoretic mobility shift assays (EMSA) to characterize binding of Sp1 and NF-κB to their respective binding sites: Sp1 −496 (Fig. 6A), Sp1 −303 (Fig. 6B), Sp1 −114 (Fig. 6C), and NF-κB −354 (Fig. 6D). EMSA revealed that incubation of the DNA–protein complexes with anti-Sp1 or anti-p65 antibodies shifted the migrating bands in an upward direction, indicating specificity for Sp1 and NF-κB (p65) proteins (Fig. 6A, 6B, 6C and 6D, lane 4). Exposure to hyperosmotic conditions for 30 min increased the binding of Sp1 to the corresponding oligonucleotides compared with the untreated control (Fig. 6A, 6B and 6C, lane 3). This indicates that hyperosmolarity increases Sp1 expression or binding of Sp1 to oligonucleotides. Similarly, as shown in Fig. 6C, hyperosmolarity treatment increases NF-κB (p65) binding to the corresponding oligonucleotides. To confirm the *in vivo* importance of Sp1 and NF-κB (p65) binding sites in response to hyperosmolarity, we performed chromatin immunoprecipitation (ChIP) analyses. As shown in Fig. 6E, under resting conditions, Sp1 binds to the I Sp1 (lane 1), II Sp1 (lane 3), and III Sp1 (lane 5) binding sites and NF-κB (p65) binds to the NF-κB-binding site (lane 7). Hyperosmolarity treatment increases the DNA-binding activities of Sp1 and NF-κB (p65) to their binding sites. Together, these results indicate that Sp1 and NF-κB binding increases under hyperosmotic conditions. These data, together with the results of the promoter studies, demonstrate the critical role of Sp1 and NF-κB in the regulation of basal and hyperosmolarity-induced SPAK expression.

### Hyperosmolarity increases SPAK expression via increased NF-κB expression *in vivo*

**Figure 7:**
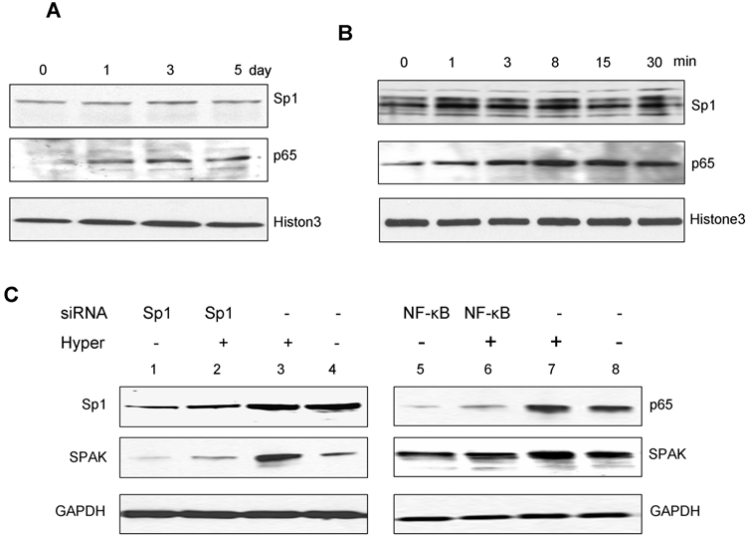
Western blots of transcription factors Sp1 and NF-κB (p65). A. Western blots of Sp1 and NF-κB (p65) demonstrating hyperosmolarity effect on Sp1 and NF-κB protein levels *in vivo*. Histone3 acts as a control. B. Western blots of Sp1 and NF-κB (p65) demonstrating hyperosmolarity effect on Sp1 and NF-κB protein levels *in vitro*. Histone3 acts as a control. C. Reduction of NF-κB but not Sp1 expression reduced SPAK protein expression in unstimulated and in hyperosmolarity-stimulated Caco2-BBE cells. Cells were harvested and subjected to western blot analysis using Sp1, NF-κB (p65), and SPAK antibodies as described in materials and methods. GAPDH acts as a loading control.

To confirm the effects of hyperosmolarity on SPAK expression, nuclear proteins were extracted from colonic epithelial cells of mice treated exposed to hyperosmotic conditions and subjected to Western-blot analysis with Sp1 and NF-κB antibodies. As shown in Fig. 7A, after different durations of hyperosmolarity exposure, there was no significant change in the Sp1 protein level; however, expression of NF-κB (p65) increased as early as the first day. These *in vivo* data show that hyperosmolarity does not affect the overall nuclear levels of Sp1; however, hyperosmolarity treatment induces translocation of NF-κB (p65) to the nucleus and upregulates its expression level. These results were confirmed with Caco2-BBE cells exposed to hyperosmotic conditions. As shown in Fig. 7B, hyperosmotic medium significantly increases NF-κB (p65) expression levels, but not Sp1 levels. Together, the *in vivo* and *in vitro* studies demonstrate that hyperosmolarity regulates SPAK in a NF-κB-dependent manner.

### Reduction of Sp1 or NF-κB expression differentially regulates SPAK expression

One approach to studying the functional role of a specific protein is to knockdown its expression. To further study SPAK regulation by Sp1 and NF-κB, we used siRNA to prevent Sp1 and NF-κB expression. As shown in Fig. 7C, Caco2-BBE cells transfected with siRNA against Sp1 and NF-κB (p65) showed decreased expression levels of Sp1 (lanes 1 and 2) and NF-κB (p65) (lanes 5 and 6) compared with Caco2-BBE cells transfected with scrambled control siRNA. Basal SPAK protein expression was also significantly reduced in Caco2-BBE cells transfected with Sp1-specific siRNA (SPAK lane 1 vs lane 4); however, there were no significant reductions in SPAK expression in cells transfected with NF-κB (p65)-specific siRNA (SPAK lane 5 vs lane 8). Thus, basal SPAK expression is effectively reduced by reduced levels of Sp1. We further investigated whether siRNA against Sp1 and/or against NF-κB reduced the levels of hyperosmolarity-induced SPAK expression. As shown in Fig. 7C, hyperosmolarity-induced SPAK expression levels were significantly reduced in Caco2-BBE cells transfected with Sp1-specific siRNA or NF-κB-specific siRNA (SPAK lanes 2 and 6, respectively) compared with cells transfected with scrambled siRNA (SPAK lane 3 and 7). These data demonstrate that Sp1 has an important role in the basal expression of SPAK, and Sp1 and NF-κB have important roles in the transcriptional regulation of SPAK expression under hyperosmotic conditions.

### SPAK expression regulates epithelial barrier function *in vitro*

**Figure 8:**
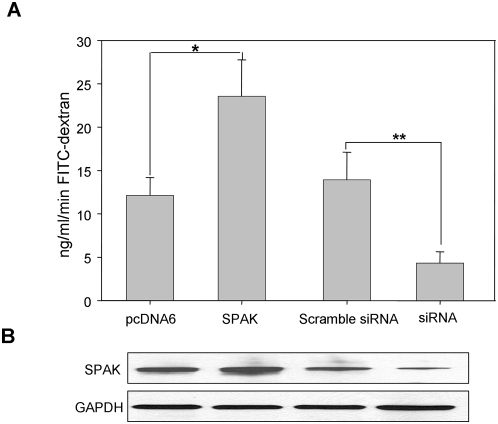
SPAK is involved in epithelial barrier function *in vitro*. A. *In vitro* permeability assay in Caco2-BBE cells transfected with pcDNA6, SPAK/pcDNA6, con siRNA or SPAK siRNA with 4 kDa FITC-Dextran. Fluorescence was quantified in lower chamber at 2 hours after the administration of FITC-dextran (λ ex = 492 nm, λ em = 510), *p<0.05, **p<0.01. B. Western blot of Caco2-BBE cells protein scraped from filter after the *in vitro* permeability assay.

On the basis of our data showing that SPAK expression is increased in colonic tissue of CD patients, we hypothesized that SPAK may play a role in epithelial barrier function. We studied permeability in Caco2-BBE cells stably transfected with SPAK/pcDNA6, vector pcDNA6, SPAK siRNA or Con siRNA using a fluorescein isothiocyanate (FITC)-labeled dextran method (Fig. 8A and 8B), as described in the *Materials and Methods*. Fluorescence was quantified in the lower chamber 2 hours after the administration of FITC–dextran. As shown in Fig. 8A, vector-transfected cells showed an FITC-dextran level of 12.1±2.6 ng of FITC/ml protein/min. In comparison, there was a ∼2-fold increase in FITC-dextran levels in SPAK/pcDNA6-transfected cells (23.8±4.03 ng of FITC/ml protein/min), almost no change in FITC–dextran levels in Con-siRNA-transfected cells (13.9±3.2 ng of FITC/ml protein/min), and a ∼3.2-fold decrease in FITC–dextran levels in SPAK-siRNA-transfected cells (4.3±1.3 ng of FITC/ml protein/min). These results indicate that overexpression of SPAK increases the permeability of cells and reduced SPAK expression decreases the permeability.

### SPAK expression regulates epithelial barrier function *in vivo*

**Figure 9:**
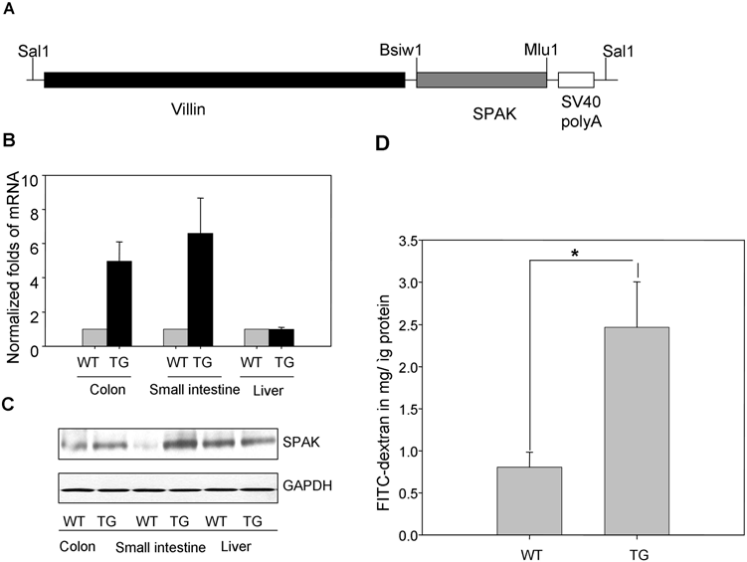
SPAK is involved in epithelial barrier function *in vivo.* A. Schematic diagram of villin/SPAK transgene construct, full length SPAK cDNA was cloned into villin vector by *Bsiw1/Mlu1* sites; villin/SPAK was digested with *Sal1* before microinjection. B. SPAK is tissue-specifically over-expressed in intestine by real time PCR with samples from small intestine, colon and liver. C. SPAK is specifically over-expressed in intestine by western blot with samples from small intestine, colon and liver. D. *in vivo* permeability assay in villin/SPAK transgenic mice, WT: wide type; TG: transgenic mice, ** p<0.01.

Transgenic mice have been widely used as a robust, dependable animal model of human disease. To better understand the function of SPAK in IECs, we generated transgenic mice that expressed a constitutively active form of SPAK under the control of the *villin* gene locus control region (LCR), which can target SPAK expression only in IECs (as shown in Fig. 9A, 9B and 9C) at the mRNA and protein levels, as described previously [40]. Our data demonstrated that Caco2-BBE cells stably transfected with SPAK showed increased permeability *in vitro*, so it was important to study the role of SPAK in epithelial barrier function in mice. With SPAK transgenic FVB/6 mice harboring the *villin* gene, which limits SPAK overexpression in the intestine, we studied barrier function in wild-type and SPAK transgenic mice using the FITC-labeled dextran method, as described in the *Materials and Methods*. Mice were administered FITC–dextran by gavage, and fluorescence was quantified in the serum 4 h after administration. As shown in Fig. 9D, wild-type mice had a FITC–dextran level of 0.805±78 mg /μg protein. By contrast, SPAK transgenic mice showed a ∼4.5-fold increase in the FITC–dextran level (3.469±234 mg /μg protein), indicating decreased barrier function in these mice.

## Discussion

In this study, we demonstrated for the first time that colonic SPAK expression levels are increased in mucosal biopsy samples of patients with CD. It is known that patients with CD have markedly higher colonic osmolarity compared with healthy individuals [1], [2], [3], and this high colonic osmolarity contributes to the activation of colonic mucosal inflammation in Crohn's colitis [1], [4]. This high osmolarity also led to increased expression of colonic SPAK in mice and a Caco2-BBE cell model, and the SPAK expression level was correlated with the extent of colonic mucosa injury. We can conclude that hyperosmolarity can upregulate the SPAK expression. Hyperosmolarity can also regulate the expression of other molecules; examples of such regulation include upregulation of yeast alpha-glycerophosphate dehydrogenase 1 (GPD1) [41], upregulation of IL-8 in bronchial epithelial cells [8], peripheral blood mononuclear cells [7] and the intestinal epithelial cell lines Caco2-BBE and HT29 [10], upregulation of the taurine transporter (TauT) in ARPE-19 cells [42], upregulation of MMP-9 in human corneal epithelial cells [6], and downregulation of the cystic fibrosis transmembrane conductance regulator (CFTR) in HT29 and T84 cells [43].

We used the SPAK TATA-less promoter to study the mechanisms underlying the hyperosmolarity-induced changes in SPAK expression during intestinal inflammation. We found that Sp1 binding sites are important both for basal and hyperosmolarity-induced promoter activities, whereas the NF-κB binding site plays an important role in the hyperosmolarity-induced promoter activity. Sp1 is known to support constitutive expression of a variety of eukaryotic genes that lack a functional TATA box and is thought to have key roles in regulating the expression of both house-keeping [44], [45] and non-house keeping genes [46]. NF-kB comprises a family of proteins that form a variety of hetero- and homodimers, the subunits of which upregulate gene transcription induced by inflammatory stimuli such as cytokines, bacterial products, viral expression, growth factors, and other stress stimuli in an NF-κB-dependent manner [47], [48]. Transcription of several genes is upregulated, including those encoding the proinflammatory cytokines IL-1, IL-6, IL-12, TNF-α, IFN-γ and IL-8, the chemokine MIP-1 and the adhesion molecule ICAM [49], [50], [51]. NFκB has also been shown to be involved in the regulation of inflammatory responses in IBD [52], [53]. NFκB is known to function together with cooperating transcription factors and coactivators, such as Sp1, to activate transcription [54], [55], [56]. For example, NF-κB can interact with Sp1 [57] to regulate HIV-1 [46], ICAM1 [58], [59], and granulocyte macrophage colony-stimulating factor 1 (GM-CSF); the interaction involves the zinc finger region of Sp1 and the N-terminal Rel homology domain of p65 [54]. Under certain conditions, NF-κB can also bind to the consensus sequence of the Sp1 binding site, thereby competing with Sp1 [60], [61].

We found that hyperosmolarity does not affect the overall nuclear levels of Sp1 in mice or Caco2-BBE cells, in agreement with previous results [62]. However, under hyperosmotic conditions the expression and translocation of NF-κB into the nucleus were increased [10], [15], [63], which indicates that increased expression of SPAK is, at least in part, due to increased expression of NF-κB as well as increased binding of Sp1 and NF-κB to their binding sites. Our data also show that knock-down of NF-κB (p65) or Sp1 by siRNA can significantly decrease SPAK expression levels, confirming their importance in the regulation of SPAK expression and indicating their potential as a target for the treatment of inflammatory diseases such as IBD [52], [64], [65], [66], [67]. Our results additionally demonstrate that NF-κB and Sp1 transcription factors are essential for regulation of SPAK expression under hyperosmotic conditions. However, SPAK expression may be regulated by multiple transcription factor pathways because we have previously reported that TNF-α-regulated SPAK expression is NF-κB-dependent but Sp1-independent [29].

Nuclear run-on assays are commonly used to determine whether an increase in mRNA level is due to an increase in transcription, a decrease in the degradation rate, or both. For example, mRNA levels in *Drosophila* [68] were found to be regulated at a post-transcriptional level without evident transcriptional regulation; however, simultaneous inhibition of both Erk and p38 kinase pathways decreased the levels of cytokine gene transcription (IL-6 and TNF-α) [69]. Our data show that exposure to hyperosmotic conditions (for 60 min) significantly increases SPAK mRNA transcription, which is in agreement with previous reports that osmolarity does not affect mRNA stability or enzyme degradation [70], [71]. However, hyperosmolarity can regulate the expression of target genes by several different mechanisms; for example, the transcriptional decrease in mRNA levels of CFTR [43] and TauT [42], and induction of IL-8 through activation of NF-κB in a p38-kinase-dependent manner [8], [10].

Hyperosmolarity has multiple and complex cellular effects. For example, hyperosmolarity decreases Na*^+^* transport and impairs barrier function of sheep rumen epithelium [72]; however, it increases the lung capillary barrier [73]. The mechanisms underlying the various aspects of barrier function affected by hyperosmolarity are complicated. First, hyperosmolarity activates the p38 and JNK pathway (Figure S1), as demonstrated previously [8], [10], [13], [43], [74], [75], which in turn can lead to increased epithelial permeability. Second, hyperosmolarity induces the production of proinflammatory cytokines such as TNF-α, IL-1β, IL-6, IL-8 (Figure S2) that disrupt epithelial barrier function, as described previously [10], [12], [13], [16], [76]. Third, hyperosmolarity can induce apoptosis, resulting in the loss of barrier function [16], [75], [77]. However, in agreement with previous reports, in the present study hyperosmolarity also activated the Erk1/2 pathway (Figure S1) [75], [78], [79], [80], which is involved in the mechanism by which permeability is decreased [81]. Furthermore, hyperosmolarity leads to redistribution and polymerization of cortical F-actin, which is involved in the downregulation of cellular permeability [82], [83], [84]. Hyperosmolarity can induce activation of focal adhesion kinase and redistribution of focal adhesion protein, which can lead to increased barrier function [85], [86]. Finally, in the present study, hyperosmolarity (610 mOsm, 30 min) did not induce significant apoptosis in Caco2-BBE cells (Figure S3), which eliminates the possibility that this pathway increases epithelial permeability.

Here we showed that upregulation of SPAK expression is directly involved in the regulation of intestinal barrier permeability. As demonstrated using the FITC–dextran method in Caco2-BBE cells and transgenic mice, SPAK can cause severe barrier dysfunction. Transgenic mice harboring the SPAK gene under control of the *villin* gene utilize the tissue-specific expression of villin in the intestinal epithelium, which facilitates transgenic SPAK expression in a tissue-specific manner [40] without causing unexpected results in other tissues. The barrier dysfunction caused by SPAK overexpression is important because it has a central role in the pathogenesis of intestinal inflammation [87], [88], [89]. Different mechanisms may underlie the SPAK-induced increase in transepithelial permeability, including activation of the p38 pathway [27], [28], which can increase epithelial permeability. A strong link has been established between the p38 pathway, cell volume change and inflammation [8], [10], [90], [91], as well as in the regulation of cell motility and wound healing [45], [92], [93].

In conclusion, we report that during inflammatory diseases (such as IBD) and hyperosmotic conditions, the SPAK expression level was significantly upregulated at the transcriptional level due to multiple factors, including the transcription factors NF-κB and Sp1. Our data demonstrate that SPAK expression is upregulated by hyperosmolarity and is an important mediator of barrier function. SPAK expression may thus contribute to the pathogenesis of intestinal inflammatory diseases such as IBD.

## Materials and Methods

### Human material

The diagnosis of IBD was based on clinical, endoscopic, and histological criteria. Clinical data for IBD patients were obtained by medical record review. Infectious colitis was ruled out by stool cultures. The collection of samples was approved by the Institutional Review Board of Emory University. Mucosal biopsy specimens from 4 Crohn's disease active patients were obtained during routine endoscopy that was performed after written informed consent was obtained. Control biopsy samples were collected from 6 volunteers undergoing colonoscopy for colorectal cancer screening who had no overt pathology including polyps. Biopsy specimens were snap frozen in Optimal Cutting Temperature immediately after endoscopic resection and stored at −80°C for histological immunostaining or homogenized to extract protein for western immunoblotting or RNA for real time PCR.

### Mouse model

C57BL/6 and FVB/6 mice (8 wk, 18–22 g) obtained from Jackson Laboratories (Bar Harbor, ME). Mice were group housed under a controlled temperature (25°C) and photoperiod (12:12-h light-dark cycle) and allowed unrestricted access to standard mouse chow and tap water. They were allowed to acclimate to these conditions for at least 7 days before inclusion in the experiments. All animal experiments were approved by The Institutional Animal Care and Use Committee of Emory University, Atlanta and were in accordance with the guide for the Care and Use of Laboratory Animal, published by the U.S. Public Health Service. Groups of mice (*n* = 6 mice/group) were treated with hyperosmotic medium (610 mOsm) prepared by dissolving mannitol (0.3 M) in phosphate buffered saline (PBS) for indicated days.

### Generation of transgenic mice

The full-length human colonic SPAK cDNA [28] spanning from nucleotide 139 to 1569 was introduced into the *BsiWI/MluI* site of pBS KS Villin MES SV40 polyA vector [40] harboring the *villin* gene, the villin promoter can target SPAK over expression in *villin*-containing intestine. The transgene SPAK digested with *SalI* were injected into the pronuclei of the fertilized eggs of the FVB/6 mice in collaboration with the Transgenic Mouse and Gene Targeting Core Facility (Emory University). Transgene-carrying mice were identified by PCR of genomic DNA extracted from mice tails with REDExtract-N-Amp Tissue PCR kit (Sigma-Aldrich, ST. Louis, MO). The primers were: Prim1F (5′ GGCTGTGATAGCACACAGGA 3′) and Prim1R (5′ CTGCCTGAACCACAGCAGTA 3′) or Prim2F (5′ TGGGTTTGCTCAGTTGAGTG 3′) and Prim2R (5′ AGTCGACGAATTCCGATTTG 3′). Stable SPAK-transgenic lines were established by backcrossing transgene-carrying founder mice with FVB/6 mice (Jackson Laboratories, Bar Harbor, ME).

### Cell culture

Human intestinal cell line Caco2-BBE was cultured according to the standard protocol. Caco2-BBE cells were treated with isosmolar medium or hyperosmotic mdium (610 mOsm) prepared by adding 0.3 M mannitol (Sigma-Aldrich, ST. Louis, MO) to regular Dulbecco's modified Eagle's medium DMEM (Invitrogen, Carlsbad, CA).

### Plasmids construction

SPAK promoter, its different truncates and site-directed mutants were cloned and constructed in our lab previously [29].

### Western blot

Western blot were carried out based on the standard methods with relevant antibodies.

### Immunohistochemistry

Immunostaining was performed according to the standard protocol. Briefly, 7-μm cryostat sections of mouse colon were fixed in buffered 4% paraformaldehyde for 30 min, blocked specimen in 5% normal rabbit serum in PBS/Triton for 1 h, and incubated with rabbit SPAK antibody (Cell signalling technology Inc, Danvers, MA) overnight at 4°C, washed with phosphate-buffered saline (PBS), and subsequently incubated with fluoresceinated secondary antibody for 1 h at room temperature. Colon sections were stained with 4′, 6-Diamidine-2′-phenylindole dihydrochloride (DAPI) to visualize nuclear.

Caco2-BBE cells grown on filters were washed and fixed with 4% paraformaldehyde in PBS with calcium for 20 min. The cells were then permeabilized with 0.1% Triton/PBS for 30 min at room temperature. Monolayers were incubated with rabbit SPAK antibody and then with relevant fluoresceinated secondary antibody same as for colon cryostat sections. Subsequently, monolayers were stained with rhodamine/phalloidin (Molecular Probes, Carlsbad, CA) to visualize actin. Samples were mounted in *p*-phenylenediamine/glycerol (1:1) and analyzed by confocal microscopy (Zeiss dual-laser confocal microscope).

### Real time PCR

total RNA from Caco2-BBE cells, mucosa of mouse colon tissue and patients biopsy specimens were extracted with TRIzol reagent (Invitrogen, Carlsbad, CA), reverse transcribed using the Thermoscript™ RT-PCR System (Invitrogen, Carlsbad, CA) and purified with the RNeasy Mini Kit (Qiagen, Germantown, MD). Real time PCRs were performed using iQ SYBR Green Supermix kit (Bio-Rad, Hercules, CA) with the iCycler sequence detection system (Bio-Rad, Hercules, CA) with specific primers. For human SPAK: sense 5′ TGGAATTAGCAACAGGAGCAGCG 3′, antisense 5′ TTTCCAAAGTGGGTGGATCATTT 3′, GAPDH acts as internal control: sense 5′ ACCACAGTCCATGCCATCAC 3′, antisense 5′ TCCACCACCCTGTTGCTGTA 3′. For mouse SPAK: sense 5′ GTAAGGCGAGTTCCTGGGTCG 3′, antisense 5′ CCAGTCGCCGTCTTCAGTCTT 3′ 36B4 acts as internal control: sense 5′ TCCAGGCTTTGGGCATCA 3′, antisense 5′ CTTTATCAGCTGCACATCACTCAGA.

### Nuclear protein extraction

Nuclear protein was extracted from Caco2-BBE cells or mucosa from mice colon tissue treated with hyperosmotic medium. Cells and mucosa were washed once in ice-cold PBS, and centrifuged at 800 rpm for 5 min. The resulting pellets were resuspended in 5 ml of cold lysis buffer (10 mM HEPES, 10 mM KC1, 1.5 mM MgC1_2_, and 0.1% Nonidet P-40; pH 7.9 at 4°C) for 10 min on ice. For the isolation of nuclei, the lysate was vigorously mixed and centrifuged for 5 min at 12,000 *g* and 4°C, and the nuclear pellet was washed once with 1 ml of Nonidet P-40-free lysis buffer. For the extraction of nuclear proteins, the nuclear pellet was resuspended in 1 ml of protein extraction buffer (420 mM NaCl, 20 mM HEPES, 1.5 MgCl_2_, 0.2 mM EDTA, and 25% glycerol; pH 7.9) for 10 min at 4°C. After being vigorous mixed, the nuclear suspension was centrifuged for 5 min at 4°C. The protein content in the final supernatant (nuclear protein extract) was measured using the Bradford method (Bio-Rad, Hercules, CA). DTT (0.5 mM), PMSF (0.5 mM), and leupeptin (10 pg/ml) were added to the lysis and extraction buffers just before use. The diluting buffer contained the same amounts of DTT and leupeptin but only 0.2 mM PMSF. Samples were stored at −70°C until use.

### Transient transfection and luciferase reporter gene assay

*Renilla* (phRL-CMV, 5 ng) and relevant SPAK promoter constructs (4 μg) were co-transfected into Caco2-BBE cells with Lipofectin (Invitrogen, Carlsbad, CA). After stimulation, the resulting luminescence was measured for 10 s in a luminometer (Luminoskan, Thermal Labsystems, MA). Each luciferase activity was normalized based on the control Renilla luciferase activity. Extracts were analyzed in triplicate, and each experiment was performed for at least three times.

### Electrophoretic Mobility Shift Assays (EMSAs)

Probes were end labeled with a Biotin 3′ End DNA Labeling Kit (Pierce, Rockford, IL). Standard EMSAs and supershift EMSAs with relevant antibodies were performed using the LightShift Chemiluminescent EMSA Kit (Pierce, Rockford, IL). Probe labeling and protocol for EMSAs were as described previously [29].

### Chromatin immunoprecipitation assay

Sp1 and NF-κB chromatin immunoprecipitation (ChIP) assays of Caco2-BBE cells treated with hyperosmolarity (610 mOsm) for 30 min were performed using the ChIP Assay Kit (Upstate Cell Signaling Solutions, Lake Placid, NY) according to the manufacturer's instructions. Briefly, Caco2-BBE cells were fixed with 1% formaldehyde for 10 minutes at 37°C to initiate cross-linking, scraped off the plate, washed with ice-cold PBS, and resuspended in 200 μl of sodium dodecyl sulfate lysis buffer for 10 minutes on ice. Cells were then sonicated with three sets of 10-second pulses at 35% power to shear the DNA into 200 1,000-bp fragments. Samples were centrifuged, and the supernatant (used as total DNA input) was diluted in ChIP dilution buffer and precleared with a protein A agarose-salmon sperm DNA slurry to reduce the nonspecific background. Samples were then immunoprecipitated with 2 μg of mouse anti-Sp1 (Upstate Cell Signaling Solutions) or 3 μg of rabbit anti-p65 antibody (Santa Cruz Biotechnology) overnight at 4°C. Complexes were collected in a protein A agarose-salmon sperm DNA slurry for 1 hour at 4°C, washed once each with the provided low-salt, high-salt, and LiCl wash buffers, and then washed twice in Tris-EDTA buffer [10 mmol/L Tris-HCl (pH 8.0) and 1 mmol/L EDTA]. The immunoprecipitated chromatin was eluted from protein A using freshly prepared elution buffer (100 mmol/L NaHCO_3_ and 1% sodium dodecyl sulfate), and the protein-DNA cross-links were reversed by treatment with NaCl (200 mmol/L) at 65°C for 4 hours. The DNA was purified by incubation with proteinase K at 45°C for 1 hour, followed by phenol-chloroform extraction and ethanol precipitation with glycogen. Sp1 (I, II, and III) and NF-κB binding sites in immunoprecipitates were detected by PCR using the following specific primers: Sp1 IF 5′ GTAAATGAACTTCAGGTTCTCTTTG 3′, Sp1 IR: 5′ CGCCCTGCGCCTTGGCCC CAGACGA 3′; Sp1 IIF 5′ AGCACACACAAAGCGGCCTGACTCC 3′, Sp1 IIR 5′ CCCAGAGCCTAGCGCGCGCTGTTCT 3′; Sp1 IIIF 5′ CTGGCTTCGGCGGGGAC GGCGGCGG 3′; Sp1 IIIR 5′ CCATGATGCTGCGGAGGAGAGCAGGAG 3′; NF-κBF 5′ GGCGCAGGGCGAGCAGGGAGGGAGG 3′, NF-κBR 5′ TGTTCTCCGCCTCGG CGAGGGGAAC 3′. The products were resolved on a 1% agarose gel and visualized with ethidium bromide.

### Transfection of siRNA

Subconfluent (60%) Caco2-BBE cells plated on six-well plates (Costar, Corning, NY) were transfected with siRNA duplexes directed against Sp1 and NF-κB, and a non targeting siRNA was used as the control for non-sequence-specific effects of siRNAs (Ambion, Austin, TX) using Lipofectamine 2000 (Invitrogen, Carlsbad, CA) in serum-free medium. Serum was added after 24 h, Caco2-BBE cells underwent hyperosmolarity treatment (610 mOsm) for 30 min; cells were then collected for western blot analysis with relevant antibodies.

### Nuclear run-on assay

Nuclear run-on assay was performed following the previous protocol [94] with little modification. Briefly; nuclei were isolated from Caco2-BBE cells treated with or without hyperosmolarity for 1 hour. Around 5×10^7^ cells were pelleted then lysed on ice for 10 min in lysis buffer containing 0.3 M sucrose, 0.4% (v/v) NP-40, 10 mM Tris-HCl at pH 7.4, 10 mM NaCl, and 3 mM MgCl_2_. After centrifugation (15 min at 500 relative centrifuge force), the nuclear pellet was resuspended and subjected to a repeat (5 min) lysis to remove any remaining intact cells. Following centrifugation, nuclei were purified by centrifugation through a 2.0 M sucrose cushion. The nuclei were resuspended in 300 μl of transcription buffer (50 mM Tris-HCl [pH 8.0], 150 mM KCl, 5 mM MgCl_2_, 0.5 mM MnCl_2_, 1 mM dithiothreitol, 0.1 mM EDTA, 10% glycerol). After pretreatment with 1 μl of 50 μg/ml RNase A and followed by 2.5 μl of 100 units RNasin, the *in vitro* elongation reaction was initiated with the addition of ribonucleotides to a final concentration of 0.25 mM each ATP, GTP, CTP and UTP. The reaction was carried out for 25 min at 30°C. After incubation with RNase-free DNase, RNA was extracted with phenol-chloroform, precipitated with ammonium acetate and isopropanol, washed with 70% ethanol, and dissolved in water. cDNA was synthesized with The SuperScript® III First-Strand Synthesis System (Invitrogen, Carlsbad, CA) and amplified with Platinum Taq DNA polymerase (Invitrogen, Carlsbad, CA) using SPAK HQF and SPAK HQR as primers. GAPDH acts as internal control. The products were resolved on a 1.5% agarose gel and visualized with ethidium bromide.

### SPAK mRNA stability assay and northern blot

For mRNA decay experiments, Caco2-BBE cells pretreated with actinomycin D (5 μg/ml) for 1 hour to arrest transcription were cultured in hyperosmotic medium (610 mOsm) or isoosmotic DMEM at indicated time points. The decay of SPAK mRNA was examined by real time PCR with specific primers SPAKHQF: 5′ TGGAATTAGCAACAGGAGCAGCG 3′ and SPAKHQR: 5′ TTTCCAAAGTGGGTGGATCATTT 3′. Levels of mRNA were then standardized against 18s rRNA levels with primers 18SF: 5′ CCCCTCGATGCTCTTAGCTGAGTGT 3′ and 18SR: 5′ CGCCGGTCCAAGAATTTCACCTCT 3′, taking into account a previous determination of 65 hours for 18s rRNA half life [95], and plotted as the percentage of remaining mRNA compared to message levels at the 0 time point (where there is a 100% maximum mRNA level). The 1-hour time point sample was also used for Northern blot analysis with the North2South complete biotin random prime labeling and detection kit (Pierce, Rockford, IL) with probe generated by PCR with primers: SPAKNorFor 5′ CTGATTGAGAAGCTGCTTACAAG 3′ and SPAKNorRev 5′ CAAGAAGAAGCTTCTCTGTAGTC 3′.

### Permeability assay

*In vitro* and *in vivo* permeability assays to determine barrier function were performed using the FITC-Dextran method as described previously [96], [97]. For *in vito* permeability assays, Caco2-BBE cells transfected with SPAK/pcDNA6, vector pcDNA6, SPAK siRNA (Ambion, Austin, TX) and con siRNA (same sequence with random order) were plated on transwell filters (Costar, Corning, New York) to grow to just confluency. For permeability assays, 100 μl of conditioned medium with FITC-Dextran (10 mg/ml, MW 4 kDa; Sigma-Aldrich, ST. Louis, MO) are added into the upper chamber of the transwell chamber, the plate is incubated at 37°C in an atmosphere of 5% CO_2_. Samples are taken from the lower chamber with/without treatment of hyperosmolarity (610 mOsm) for 30 min. The permeability of the epithelial monolayer correlates with the fluorescence intensity in the lower chamber (λ ex = 492 nm, λ em = 510 nm; Cytofluor 2300; Millipore, Waters Chromatography). Values are shown as nanograms per milliliter per minute FITC-dextran present in the basolateral reservoir. For *in vivo* permeability assay, 8-wk old wild type and SPAK transgenic mice were used. Food and water were withdrawn for 4 h and mice were gavaged with permeability tracer FITC-labeled dextran (60 mg/100 g body weight MW 4 kDa; Sigma-Aldrich, ST. Louis, MO). Serum was collected retro-orbitally 4 h after gavage; fluorescence intensity of each sample was measured (λ ex = 492 nm, λ em = 510 nm; Cytofluor 2300; Millipore, Waters Chromatography); and FITC-dextran concentrations were determined from standard curves generated by serial dilution of FITC-dextran and represented as μg FITC-dextran/μg protein.

## Supporting Information

Figure S1 (0.65 MB DOC)

Figure S2 (0.03 MB DOC)

Figure S3 (0.27 MB DOC)
