# Supplementary material for: Ste20-Related Proline/Alanine-Rich Kinase (SPAK) Regulated Transcriptionally by Hyperosmolarity Is Involved in Intestinal Barrier Function
Source: PLoS One. 2009 Apr 3;4(4):e5049. doi: 10.1371/journal.pone.0005049 (PMC2660421; doi:10.1371/journal.pone.0005049)
Supplement: Figure S1 — (0.65 MB DOC) [file pone.0005049.s001.doc]

**Figure S1.** Hyperosmolarity leads to activation of mitogen activated protein kinases (MAPK). The evolutionarily conserved MAPK signaling pathway plays an important role in transducing signals from diverse extracellular stimuli (including growth factors, cytokines and environmental stresses) to the nucleus to affect a wide range of cellular processes, such as proliferation, differentiation, development, stress responses and apoptosis. To study the effect of hyperosmolarity on MAPK pathways, Caco2-BBE cells were plated on 6 well plates, and grow until confluent, and treated with isosmolar medium or hyperosmolar mdium (610 mOsm) prepared by dissolving 0.3 M mannitol (Sigma-Aldrich, ST. Louis, MO) in regular Dulbecco’s modified Eagle’s medium DMEM (Invitrogen, Carlsbad, CA) at the indicated time. The wells were then lyzed with RIPA buffer supplemental with NaVO4, NaF, after collection, centrifugation, and equal mount of supernatant was subjected to western blots with relevant antibodies: phospho-Erk1/2, total Erk1/2, phospho-p38, total p38, phospho-JNK, total JNK (Cell signalling technology Inc, Danvers, MA), Histone3 (Millipore, Billerica, MA). Hyperosmolarity can phosphorate Erk1/2, JNK and p38, indicating hypersomolarity is involved in variety of different processes or functions. Furthermore, hyperosmolarity can increase the expression of total JNK, not total Erk1/2 and p38.
