# Supplementary material for: Ste20-Related Proline/Alanine-Rich Kinase (SPAK) Regulated Transcriptionally by Hyperosmolarity Is Involved in Intestinal Barrier Function
Source: PLoS One. 2009 Apr 3;4(4):e5049. doi: 10.1371/journal.pone.0005049 (PMC2660421; doi:10.1371/journal.pone.0005049)
Supplement: Figure S2 — (0.03 MB DOC) [file pone.0005049.s002.doc]

**Figure S2.** Hyperosmolarity increases the production of proinflammatory cytokines. Proinflammatory cytokines play a central role in the pathogenesis of IBD including Crohn`s diseases, there is hyperosmotic environment in colon in patients with Crohn`s disease. We hypothesis that hyperosmolarity might be involved in the production of proinflammatory cytokines. To verify this hypothesis, we employed real time PCR to study the proinflammatory cytokines production under the hyperosmolarity treatment in Caco2-BBE cell. Caco2-BBE cells were plated on 6 cell plates, and grow until confluent, and treated with isosmolar medium or hyperosmolar mdium (610 mOsm) prepared by dissolving 0.3 M mannitol (Sigma-Aldrich, ST. Louis, MO) in regular Dulbecco’s modified Eagle’s medium (DMEM, Invitrogen, Carlsbad, CA) at the indicated time. Real time PCRs were performed using iQ SYBR Green Supermix kit (BioRad, Hercules, CA) with the iCycler sequence detection system (BioRad, Hercules, CA). Specific primers to measure the production of different cytokines TNF-α: TNF-α sense 5’ AGG CTG CCC CGA CTA CGT 3’, antisense 5’ GAC TTT CTC CTG GTA TGA GAT AGC AAA 3’; IL-1β: sense 5’ TCG CTC AGG GTC ACA AGA AA 3’, antisense 5’ CAT CAG AGG CAA GGA GGA AAA C 3’; IL-6: sense 5’ ACA AGT CGG AGG CTT AAT TAC ACA T 3’, antisense 5’ TTG CCA TTG CAC AAC TCT TTT C 3’ and IL-8: sense 5’ GTG CAG TTT TGC CAA GGA GT 3’, antisense 5’ AAA TTT GGG GTG GAA AGG TT 3’, and GAPDH: sense 5’ GTC GGA GTC AAC GGA TTT GG 3’, antisense 5’ AAG CTT CCC GTT CTC AGC CT 3’. As shown in Figure S2. hyperosmolarity can increase the production significantly of proinflammatory cytokines TNF-α, IL-1β, IL-6 and IL-8. Furthermore, this stimulation happened very early after the treatment, for example, IL-1β and IL-6 production increase as early as 3 minutes. This data can bring about a link among hyperosmolarity, proinflammatory cytokines and inflammatory bowel diseases.
