# Supplementary material for: Ste20-Related Proline/Alanine-Rich Kinase (SPAK) Regulated Transcriptionally by Hyperosmolarity Is Involved in Intestinal Barrier Function
Source: PLoS One. 2009 Apr 3;4(4):e5049. doi: 10.1371/journal.pone.0005049 (PMC2660421; doi:10.1371/journal.pone.0005049)
Supplement: Figure S3 — (0.27 MB DOC) [file pone.0005049.s003.doc]

**Figure S3. Hyperosmolarity (610 mOSM) induces no significant apoptosis**

It has been shown that with different doses of hyperosmolarity, with different time treatment of hyperosmolarity, in different tissue or cell line, hyperosmolarity can cause obvious apoptosis. So it is very crucial to determine if this dosage of hyperosmolarity (610 mOsm) can induce apoptosis in Caco2-BBE cells at 30 min. Therefore, we performed western blots with caspase3 antibody. Caco2-BBE cells were plated on 6 cell plates, and grow until confluent, and treated with isosmolar medium or hyperosmolar mdium (610 mOsm) prepared by dissolving 0.3 M mannitol (Sigma-Aldrich, ST. Louis, MO) in regular Dulbecco’s modified Eagle’s medium DMEM (Invitrogen, Carlsbad, CA) at the indicated time. The cells were then lyzed with RIPA buffer supplemental with NaVO4, NaF, after collection, centrifugation, and equal mount of supernatant was subjected to western blot with Caspase 3 (Cell signalling technology Inc, Danvers, MA). We could not find the cleavage of caspase3 either, which means no significant apoptosis involved, at least during the 30 min treatment of hyperosmolarity.
